# Correlation between fruit consumption and 10-year all-cause mortality in patients with dyslipidemia

**DOI:** 10.3389/fnut.2024.1471737

**Published:** 2024-10-03

**Authors:** Yuanjuan Zheng, Feifei Sun, Suling Ye, Jinzhou Zhu, Yu Ma, Mengmeng Shan, Shaomi Li, Yingying Chen, Jie Li

**Affiliations:** ^1^Department of General Practice, The First Affiliated Hospital of Yangtze University, Jingzhou, China; ^2^Department of Critical Care Medicine, Heilongjiang Provincial Corps Hospital of Chinese People’s Armed Police Forces, Harbin, China; ^3^Department of Internal Medicine, Ruijin-Hainan Hospital Shanghai Jiao Tong University School of Medicine (Hainan Boao Research Hospital), Qionghai, China

**Keywords:** fruit, apple, banana, dyslipidemia, mortality, NHANES

## Abstract

**Background:**

Consuming fruit provides health benefits. Reportedly, increased fruit consumption reduces the risks of hypertension and cardiovascular disease. However, existing studies have not clarified the effect of fruit consumption on mortality risk in patients with dyslipidemia. This study aimed to assess the correlation between the consumption of different types of fruits and all-cause mortality in patients with dyslipidemia.

**Methods:**

A total of 2,184 patients with dyslipidemia were included in this study, and trends in the correlation between the frequency of consumption of different types of fruits and the 10-year all-cause mortality risk in patients with dyslipidemia were analyzed by smoothed curve fitting, Cox regression, and Kaplan–Meier curve analysis. Subgroup analysis and interaction test were applied to analyze the stability of the effect of apple consumption on 10-year all-cause mortality in patients with dyslipidemia.

**Results:**

Smoothed curve fitting and Cox regression analyses revealed a significant reduction in the 10-year all-cause mortality risk in patients with dyslipidemia who consumed apples 3–4 times/week (hazard ratio [HR] = 0.61, 95% confidence interval [CI]: 0.43–0.87, *p* = 0.007) and in those who consumed bananas 3–4 times/week (HR = 0.71, 95% CI: 0.52–0.98, *p* = 0.039), with a more pronounced effect in patients who consumed both apples and bananas (HR = 0.55, 95% CI: 0.30–0.99, *p* = 0.045). Other fruits did not exhibit similar effects.

**Conclusion:**

Consuming apples or bananas 3–4 times/week significantly improved the 10-year survival rate in patients with dyslipidemia, and the effect was even more profound in patients who consumed both fruits.

## Introduction

Dyslipidemia is a common metabolic disorder characterized by elevated serum cholesterol or triglyceride levels (1). Dyslipidemia affects more than 100 million adults in the United States, and it has a prevalence rate of 53%. Moreover, the prevalence rates of hypercholesterolemia (13%) and hypertriglyceridemia (10.7%) in children and adolescents are on the rise (2). Dyslipidemia leads to endothelial cell damage and dysfunction, which is the basis for the development and progression of atherosclerosis (3) and one of the important risk factors for the development of cardiovascular diseases. In particular, the incidence and mortality rates of cardiovascular and cerebrovascular diseases increase markedly in patients with dyslipidemia combined with hypertension or diabetes mellitus (4). Therefore, proactive and effective intervention for patients with dyslipidemia is an important measure to improve their health and reduce their risk of cardiovascular and cerebrovascular mortality.

Optimizing diet is an important lifestyle intervention for managing dyslipidemia and cardiovascular disease (5), and fruits are an important component of a healthy diet. Micronutrients and polyphenolic compounds in fruits are known to promote human health (6). Dietary fiber, vitamins, flavonoids, sterols, and other antioxidant compounds can lower blood lipids, inhibit low-density lipoprotein (LDL) oxidation, eliminate oxygen free radicals, and ameliorate dyslipidemia through boosting the immune system and improving metabolic disorders (4). Low fruit consumption is considered to be the fifth largest contributor to the global burden of disease (7). Therefore, most nutritionists recommend that adults consume at least two servings of fruit per day (8). The World Health Organization also recommends increased fruit intake in patients with dyslipidemia but does not specify the exact type of fruit to be consumed.

Reportedly, moderate apple and banana intake reduces the risk of all-cause mortality in patients with hypertension (9). However, there are no studies that have analyzed the effect of fruit consumption on mortality in patients with dyslipidemia. In order to provide scientific basis for clinicians, so that they can formulate appropriate fruit consumption strategies for patients with dyslipidemia, thereby reducing the risk of all-cause mortality in patients with dyslipidemia. Therefore, this study investigated the effect of fruit intake on the prognosis of patients with dyslipidemia using the National Health and Nutrition Examination Survey database (NHANES).

## Methods

### General data

The study data were obtained from NHANES, and all data are available on the NHANES official website.^1^

**Figure 1:**
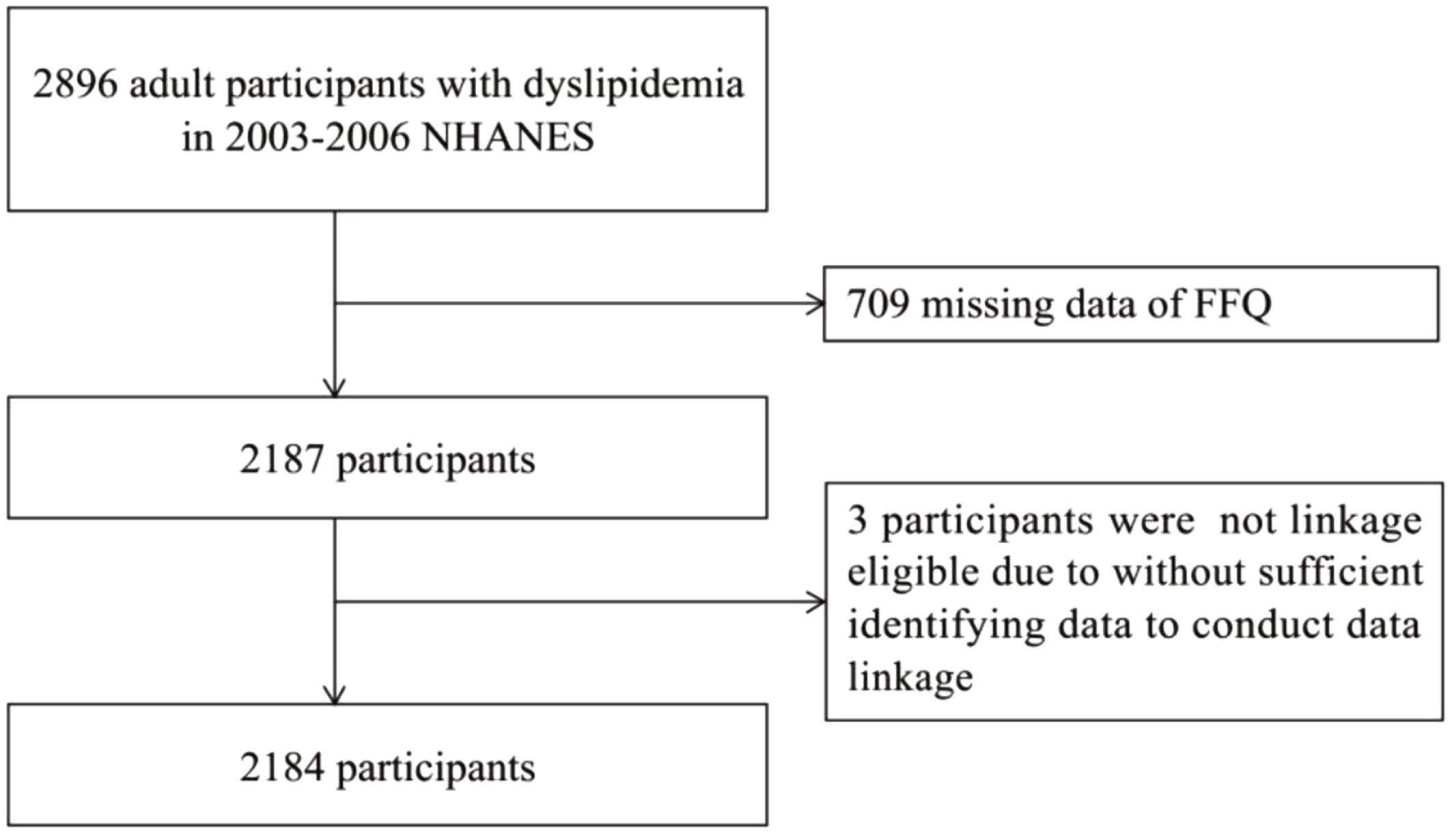
Flowchart of participant of selection.

Between 2003 and 2006, a total of 2,896 adults with dyslipidemia from diverse racial and ethnic backgrounds and regions were included in NHANES. Among them, 709 adults were excluded from this study because of missing data from the food frequency questionnaire (FFQ). In addition, three adults, who did not have National Death Index (NDI) data records, were excluded because of missing follow-up results on survival status. Ultimately, a total of 2,184 participants were included in this study (Figure 1).

### Fruit consumption assessment

The FFQ was designed as an instrument to collect information on the frequency of food intake by participants, including fruit consumption habits, over the past 12 months. For different kinds of fruits, participants will answer the frequency of intake in the past year. In this study, the fruits with specific intake frequency information were included, including apple, banana, pineapple and grape. The intake levels were categorized as follows: unknown, < 1 time/month, 1–3 times/month, 1–2 times/week, 3–4 times/week, 5–6 times/week, and ≥ 1 time/day.

### Survival status and duration of follow-up

Follow-up data were obtained from the NDI death certificate records from the publicly available file on the official website.

### Analysis of baseline data

The baseline data of the participants were obtained through a computer-assisted personal interview system and questionnaires. The data included sex, age, race, education level, ratio of household income to poverty threshold, muscle strengthening activities, and history of smoking, alcohol consumption, hypertension, diabetes, heart failure, coronary heart disease, and stroke. Hypertension, diabetes, heart failure, and stroke were defined as having been diagnosed with these conditions by a doctor. A participant was defined as a patient with coronary heart disease if he or she answered “yes” to one of the following three questions: “Have you ever been told by a doctor that you have coronary heart disease/angina/heart attack?”

### Statistical methods

Continuous variables are here given as the median (first quartile, third quartile), while categorical variables are summarized as numbers with percentages. Differences in categorical variables between the survival and death groups were compared using the chi-square test, and differences in continuous variables between groups were compared using the rank sum test. Trends in the correlation between frequency of consuming different types of fruits and 10-year all-cause mortality in patients with dyslipidemia were analyzed by smoothed curve fitting, which based on a restricted cubic spline function. The effect of fruit consumption on 10-year all-cause mortality was analyzed by Cox regression, and the consistency of the results in the presence of comorbidities was verified by stratified analysis. Kaplan–Meier (KM) curves and three-dimensional histograms were plotted based on the results of Cox regression. Before regression analysis, all covariates underwent collinearity screening, with none exhibiting a variance inflation factor exceeding 5. Three regression models were established to systematically adjust for confounding factors. Model 1 remained unadjusted for confounders, while Model 2 included adjustments for age, gender, and race. In Model 3, in addition to the fixed demographic adjustments, further covariates were selected based on the principle of covariate screening. Specifically, a confounding factor was included in the regression model if the change in *p*-values exceeded 10% when comparing before and after its introduction into the model. Ultimately, the adjustment factors in Model 3 comprised gender, age, race, education level, ratio of family income to the poverty, muscle strengthening activities, smoking, hypertension, diabetes, heart failure, coronary heart disease and stroke. EmpowerStats^2^ was applied to analyze the data in this study, and *p* < 0.05 was used to indicate statistically significant results.

## Results

### Baseline data of the participants

A total of 2,184 adult patients with dyslipidemia (1,722 in the survival group, and 464 in the death group) were included in this study. The specific data of the participants are shown in Table 1.

**Table 1:** Baseline characteristics of participants.

	All	Survival	All-case death	p
Number	2,184	1722	462	
Age	62.0 (49.0–72.0)	59.0 (46.0–68.0)	75.0 (66.0–80.0)	<0.001
Sex				<0.001
Male	1,040 (47.6)	784 (45.5)	256 (55.4)	
Female	1,144 (52.4)	938 (54.5)	206 (44.6)	
Race				0.002
Mexican American	341 (15.6)	286 (16.6)	55 (11.9)	
Other Hispanic	56 (2.6)	48 (2.8)	8 (1.7)	
Non-Hispanic White	1,372 (62.8)	1,044 (60.6)	328 (71.0)	
Non-Hispanic Black	346 (15.8)	286 (16.6)	60 (13.0)	
Other race	69 (3.2)	58 (3.4)	11 (2.4)	
Education level				<0.001
<High school	560 (25.6)	390 (22.6)	170 (36.8)	
High school graduate or general equivalency diploma	590 (27.0)	463 (26.9)	127 (27.5)	
>High school	1,032 (47.3)	869 (50.5)	163 (35.3)	
Unknown	2 (0.1)	0 (0.0)	2 (0.4)	
Ratio of family income to poverty				<0.001
≤1	255 (11.7)	188 (10.9)	67 (14.5)	
1–3	936 (42.9)	684 (39.7)	252 (54.5)	
>3	890 (40.8)	772 (44.8)	118 (25.5)	
Unknown	103 (4.7)	78 (4.5)	25 (5.4)	
Muscle strengthening activities				<0.001
Yes	506 (23.2)	439 (25.5)	67 (14.5)	
No	1,597 (73.1)	1,238 (71.9)	359 (77.7)	
Unable to do activity	81 (3.7)	45 (2.6)	36 (7.8)	
Alcohol use				0.105
No	767 (35.1)	590 (34.3)	177 (38.3)	
Yes	1,417 (64.9)	1,132 (65.7)	285 (61.7)	
Smoking				<0.001
No	1,005 (46.0)	839 (48.7)	166 (35.9)	
Yes	1,179 (54.0)	883 (51.3)	296 (64.1)	
Hypertension				<0.001
No	977 (44.7)	860 (49.9)	117 (25.3)	
Yes	1,207 (55.3)	862 (50.1)	345 (74.7)	
Diabetes				<0.001
No	1768 (81.0)	1,443 (83.8)	325 (70.3)	
Yes	416 (19.0)	279 (16.2)	137 (29.7)	
Heart Failure				<0.001
No	2045 (93.6)	1,664 (96.6)	381 (82.5)	
Yes	139 (6.4)	58 (3.4)	81 (17.5)	
Cardiovascular Disease				<0.001
No	1824 (83.5)	1,522 (88.4)	302 (65.4)	
Yes	360 (16.5)	200 (11.6)	160 (34.6)	
Stroke				<0.001
No	2037 (93.3)	1,658 (96.3)	379 (82.0)	
Yes	147 (6.7)	64 (3.7)	83 (18.0)	
Apple				0.016
<1 time/month	799 (36.6)	603 (35.0)	196 (42.4)	
1–3 times/month	560 (25.6)	458 (26.6)	102 (22.1)	
1–2 times/week	371 (17.0)	300 (17.4)	71 (15.4)	
3–4 times/week	235 (10.8)	194 (11.3)	41 (8.9)	
5–6 times/week	73 (3.3)	58 (3.4)	15 (3.2)	
≧1 time/day	109 (5.0)	85 (4.9)	24 (5.2)	
Unknown	37 (1.7)	24 (1.4)	13 (2.8)	
Banana				0.002
<1 time/month	448 (20.5)	361 (21.0)	87 (18.8)	
1–3 times/month	454 (20.8)	374 (21.7)	80 (17.3)	
1–2 times/week	432 (19.8)	347 (20.2)	85 (18.4)	
3–4 times/week	397 (18.2)	315 (18.3)	82 (17.7)	
5–6 times/week	153 (7.0)	116 (6.7)	37 (8.0)	
≧1 time/day	255 (11.7)	178 (10.3)	77 (16.7)	
Unknown	45 (2.1)	31 (1.8)	14 (3.0)	
Pineapple				0.584
<1 time/month	1,480 (67.8)	1,157 (67.2)	323 (69.9)	
1–3 times/month	478 (21.9)	387 (22.5)	91 (19.7)	
1–2 times/week	133 (6.1)	107 (6.2)	26 (5.6)	
3–4 times/week	30 (1.4)	25 (1.5)	5 (1.1)	
5–6 times/week	8 (0.4)	7 (0.4)	1 (0.2)	
≧1 time/day	14 (0.6)	10 (0.6)	4 (0.9)	
Unknown	41 (1.9)	29 (1.7)	12 (2.6)	
Grape				0.404
<1 time/month	961 (44.0)	739 (42.9)	222 (48.1)	
1–3 times/month	664 (30.4)	536 (31.1)	128 (27.7)	
1–2 times/week	292 (13.4)	231 (13.4)	61 (13.2)	
3–4 times/week	109 (5.0)	90 (5.2)	19 (4.1)	
5–6 times/week	53 (2.4)	44 (2.6)	9 (1.9)	
≧1 time/day	51 (2.3)	42 (2.4)	9 (1.9)	
Unknown	54 (2.5)	40 (2.3)	14 (3.0)	

### Analysis of correlation between fruit intake and 10-year all-cause mortality in patients with dyslipidemia

**Figure 2:**
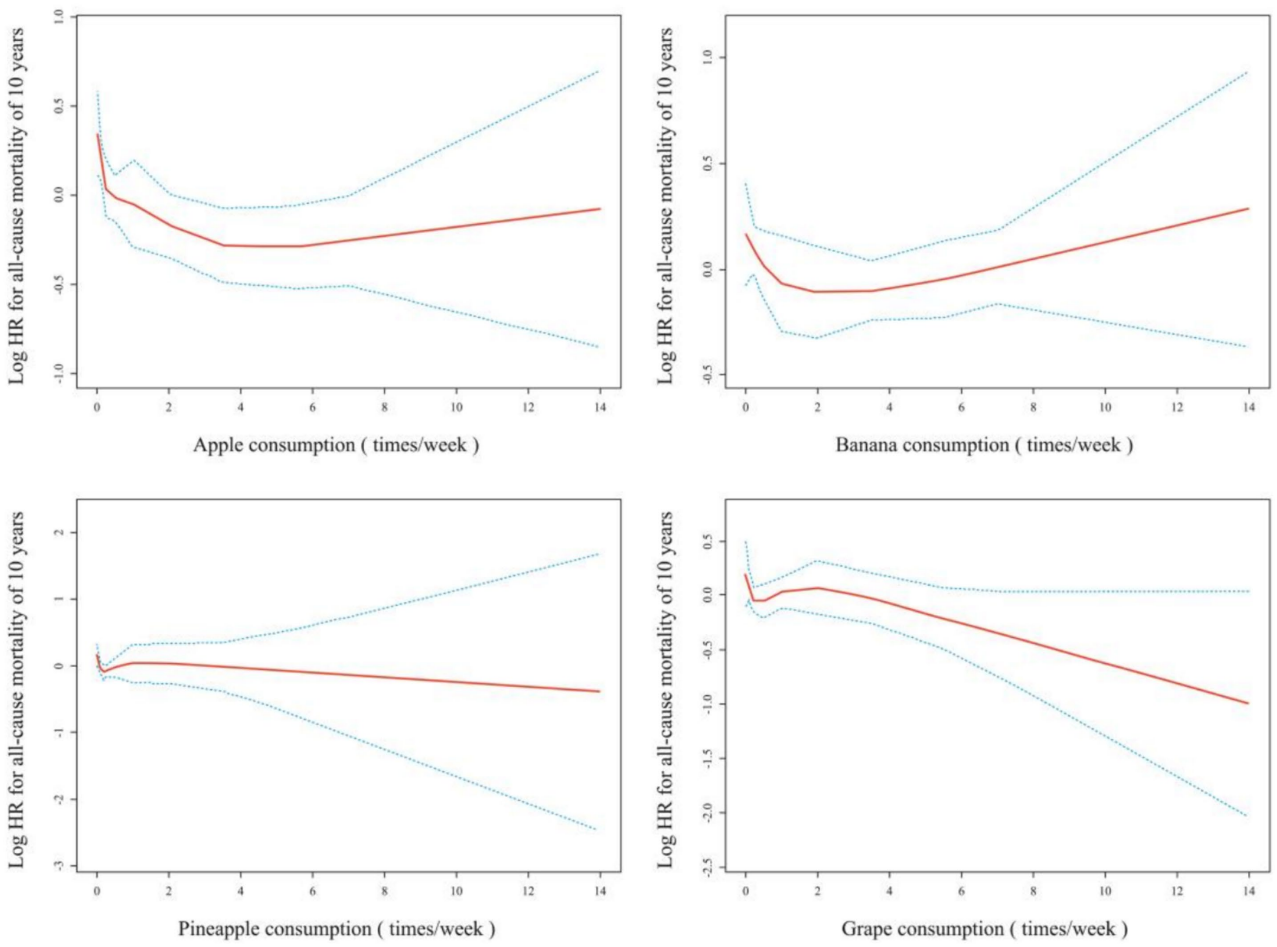
Spline smoothing plots between fruit consuming and all-cause mortality. Adjusted for gender, age, race, education level, ratio of family income to the poverty, muscle strengthening activities, smoking, hypertension, diabetes, heart failure, coronary heart disease and stroke.

The smoothing curves demonstrated that an appropriate intake of apples and bananas reduced the 10-year all-cause mortality risk in patients with dyslipidemia (Figure 2). The results of Cox regression suggested that consuming apples or bananas 3–4 times/week significantly reduced the 10-year all-cause mortality risk in patients with dyslipidemia after fully adjusting for confounders (hazard ratio [HR] = 0.61, 95% confidence interval [CI]: 0.43–0.87, *p* = 0.007 for apples; HR = 0.71, 95% CI: 0.52–0.98, *p* = 0.039 for bananas). Although the results of smoothed curve fitting and Cox regression analyses suggested that consuming pineapple or grapes also reduced the 10-year all-cause mortality in patients with dyslipidemia, the reduction was not significant (Table 2).

**Table 2:** Cox regression analysis.

	Number	Model 1	Model 2	Model 3
		HR (95%CI) P	HR (95%CI) P	HR (95%CI) P
Apple				
<1 time/month	799	Ref.	Ref.	Ref.
1–3 times/month	560	0.72 (0.57, 0.92) 0.008	0.81 (0.64, 1.04) 0.094	0.92 (0.71, 1.18) 0.493
1–2 times/week	371	0.76 (0.58, 1.00) 0.047	0.70 (0.54, 0.93) 0.012	0.77 (0.58, 1.02) 0.068
3–4 times/week	235	0.69 (0.49, 0.97) 0.031	0.56 (0.40, 0.79) <0.001	0.61 (0.43, 0.87) 0.007
5–6 times/week	73	0.83 (0.49, 1.40) 0.475	0.60 (0.35, 1.01) 0.053	0.68 (0.39, 1.18) 0.168
≧1 time/day	109	0.89 (0.58, 1.36) 0.579	0.62 (0.40, 0.95) 0.027	0.77 (0.49, 1.19) 0.235
Banana				
<1 time/month	448	Ref.	Ref.	Ref.
1–3 times/month	454	0.88 (0.65, 1.19) 0.417	0.85 (0.62, 1.15) 0.280	0.81 (0.59, 1.12) 0.206
1–2 times/week	432	1.00 (0.74, 1.35) 0.983	0.82 (0.61, 1.11) 0.197	0.78 (0.57, 1.07) 0.121
3–4 times/week	397	1.06 (0.79, 1.44) 0.692	0.72 (0.53, 0.97) 0.033	0.71 (0.52, 0.98) 0.039
5–6 times/week	153	1.27 (0.86, 1.86) 0.227	0.75 (0.51, 1.10) 0.141	0.79 (0.53, 1.17) 0.242
≧1 time/day	255	1.67 (1.23, 2.27) 0.001	0.84 (0.61, 1.14) 0.258	0.96 (0.69, 1.32) 0.786
Pineapple				
<1 time/month	1,480	Ref.	Ref.	Ref.
1–3 times/month	478	0.86 (0.68, 1.09) 0.217	0.87 (0.69, 1.10) 0.239	0.94 (0.74, 1.19) 0.586
1–2 times/week	133	0.92 (0.61, 1.37) 0.670	0.98 (0.66, 1.47) 0.935	0.95 (0.62, 1.45) 0.803
3–4 times/week	30	0.75 (0.31, 1.82) 0.525	0.84 (0.35, 2.03) 0.699	1.18 (0.48, 2.86) 0.722
5–6 times/week	8	0.55 (0.08, 3.91) 0.550	1.31 (0.18, 9.36) 0.788	1.29 (0.18, 9.21) 0.803
≧1 time/day	14	1.30 (0.49, 3.49) 0.599	0.94 (0.35, 2.55) 0.907	0.75 (0.27, 2.13) 0.593
Grape				
<1 time/month	961	Ref.	Ref.	Ref.
1–3 times/month	664	0.83 (0.67, 1.03) 0.092	0.79 (0.64, 0.98) 0.036	0.87 (0.69, 1.09) 0.217
1–2 times/week	292	0.90 (0.68, 1.19) 0.463	0.92 (0.69, 1.22) 0.540	1.07 (0.80, 1.44) 0.634
3–4 times/week	109	0.74 (0.47, 1.19) 0.215	0.73 (0.46, 1.17) 0.194	0.87 (0.53, 1.41) 0.567
5–6 times/week	53	0.72 (0.37, 1.40) 0.332	0.60 (0.31, 1.17) 0.134	0.54 (0.27, 1.11) 0.092
≧1 time/day	51	0.73 (0.38, 1.43) 0.365	0.59 (0.30, 1.16) 0.1246	0.65 (0.32, 1.33) 0.239

Model 1: Not adjusted. Model 2: Adjusted for gender, age, race. Model 3: Adjusted for gender, age, race, education level, ratio of family income to the poverty, muscle strengthening activities, smoking, hypertension, diabetes, heart failure, coronary heart disease and stroke.

### Analysis of correlation between apple intake and 10-year all-cause mortality in patients with dyslipidemia

**Figure 3:**
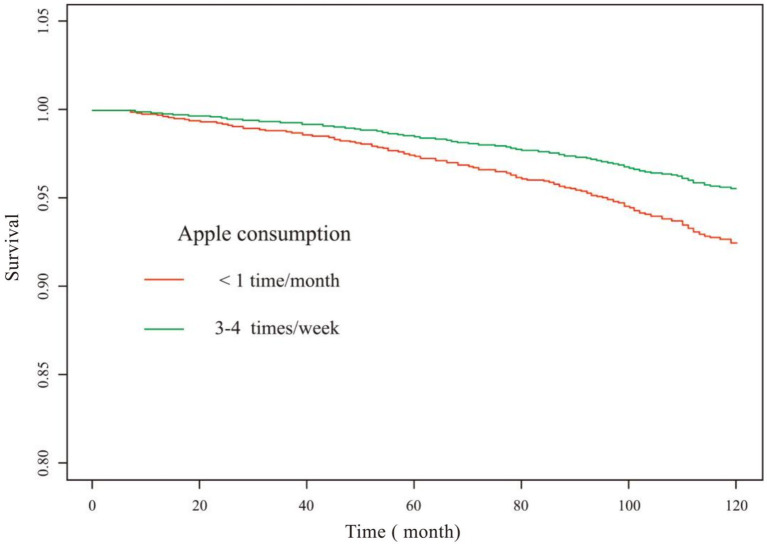
KM survival curve (apple consumption). Adjusted for gender, age, race, education level, ratio of family income to the poverty, muscle strengthening activities, smoking, hypertension, diabetes, heart failure, coronary heart disease and stroke.

Subgroup analyses and interaction tests revealed no effect modifiers (Table 3). The KM curves suggested that, compared with patients with dyslipidemia who consumed apples less than 1 time/month, those who consumed apples 3–4 times/week exhibited a significantly improved 10-year survival (Figure 3).

**Table 3:** Stratified analysis (apple consumption).

	Number	HR (95%CI)						P interaction
		<1 times/month	1–3 times/month	1–2 times/week	3–4 times/week	5–6 times/week	≧1 times/day	
Hypertension								0.240
No	977	Ref.	1.08 (0.64, 1.83)	0.96 (0.51, 1.78)	0.84 (0.43, 1.63)	0.94 (0.36, 2.46)	0.18 (0.04, 0.78)	
Yes	1,207	Ref.	0.87 (0.65, 1.16)	0.77 (0.56, 1.06)	0.50 (0.33, 0.78)	0.64 (0.32, 1.26)	0.97 (0.61, 1.55)	
Diabetes								0.627
No	1768	Ref.	0.96 (0.71, 1.30)	0.79 (0.56, 1.12)	0.73 (0.48, 1.11)	0.53 (0.26, 1.10)	0.93 (0.53, 1.61)	
Yes	416	Ref.	0.84 (0.52, 1.34)	0.80 (0.48, 1.35)	0.38 (0.18, 0.77)	0.85 (0.35, 2.04)	0.71 (0.34, 1.48)	
Heart Failure								0.226
No	2045	Ref.	0.84 (0.64, 1.12)	0.75 (0.54, 1.03)	0.65 (0.44, 0.95)	0.76 (0.43, 1.35)	0.74 (0.46, 1.20)	
Yes	139	Ref.	1.64 (0.85, 3.16)	1.14 (0.59, 2.20)	0.57 (0.18, 1.86)	0.24 (0.03, 1.81)	1.41 (0.41, 4.82)	
Cardiovascular Disease								0.619
No	1824	Ref.	0.94 (0.69, 1.28)	0.66 (0.46, 0.95)	0.64 (0.42, 0.98)	0.75 (0.37, 1.55)	0.76 (0.44, 1.32)	
Yes	360	Ref.	0.87 (0.55, 1.39)	1.05 (0.65, 1.70)	0.51 (0.25, 1.04)	0.70 (0.30, 1.65)	0.82 (0.38, 1.78)	
Stroke								0.540
No	2037	Ref.	0.91 (0.69, 1.21)	0.73 (0.53, 1.01)	0.60 (0.41, 0.88)	0.69 (0.39, 1.23)	0.97 (0.60, 1.56)	
Yes	147	Ref.	0.68 (0.35, 1.33)	1.00 (0.51, 1.96)	0.69 (0.22, 2.12)	0.46 (0.06, 3.74)	0.51 (0.16, 1.57)	

Adjusted for gender, age, race, education level, ratio of family income to the poverty, muscle strengthening activities, smoking, hypertension, diabetes, heart failure, coronary heart disease and stroke (in addition to the stratification factor itself).

### Analysis of correlation between banana intake and 10-year all-cause mortality in patients with dyslipidemia

**Figure 4:**
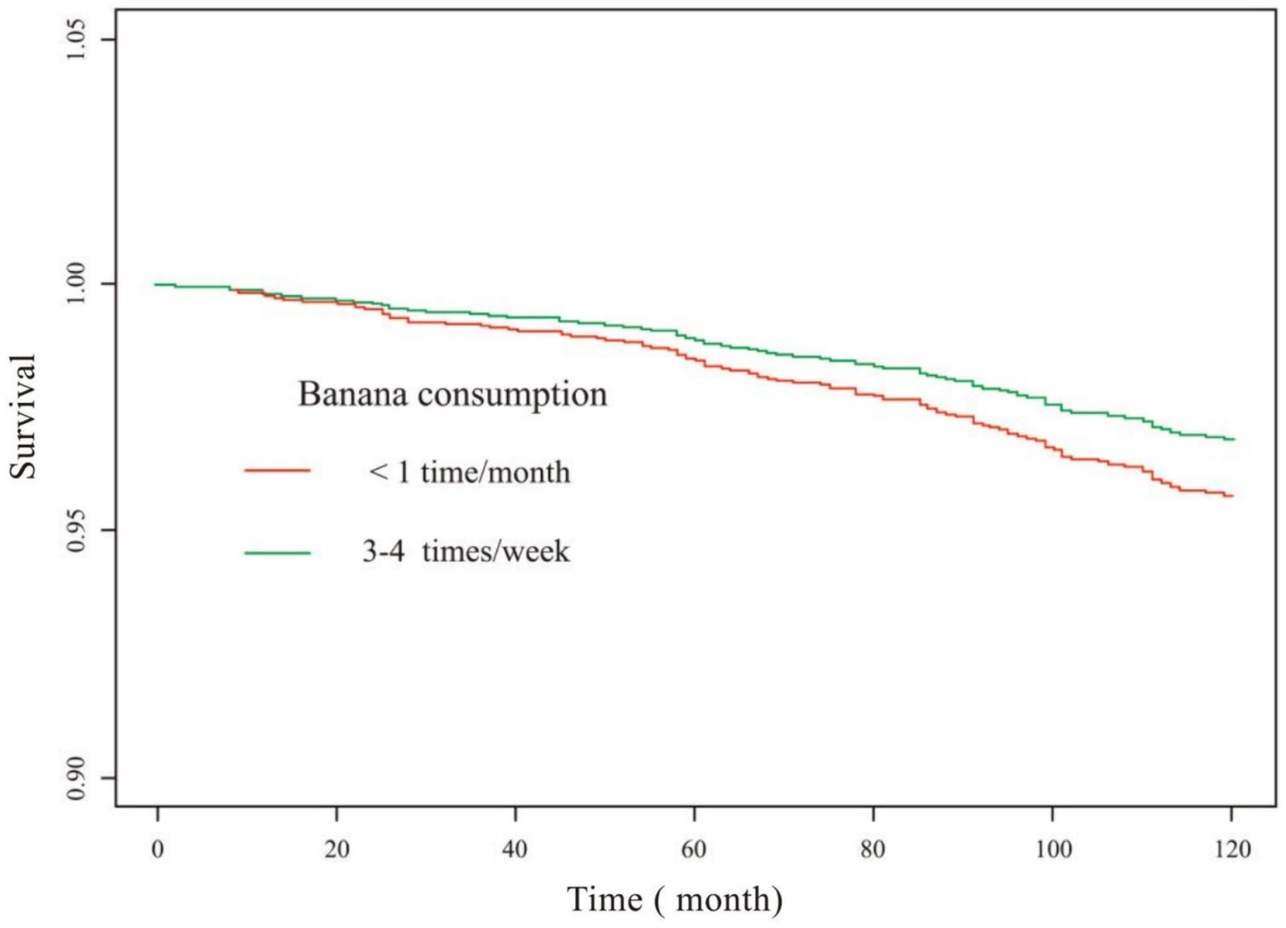
KM survival curve (banana consumption). Adjusted for gender, age, race, education level, ratio of family income to the poverty, muscle strengthening activities, smoking, hypertension, diabetes, heart failure, coronary heart disease and stroke.

The correlation between banana intake and risk of dyslipidemia-associated mortality was independent of comorbidities (Table 4). The KM curves suggested that consuming bananas 3–4 times/week significantly improved 10-year survival in patients with dyslipidemia (Figure 4).

**Table 4:** Stratified analysis (banana consumption).

	Number	HR (95%CI)						P interaction
		<1 time/month	1–3 times/month	1–2 times/week	3–4 times/week	5–6 times/week	≧1 time/day	
Hypertension								0.662
No	977	Ref.	0.90 (0.44, 1.85)	1.17 (0.58, 2.37)	0.97 (0.47, 2.00)	1.42 (0.60, 3.33)	0.89 (0.43, 1.86)	
Yes	1,207	Ref.	0.81 (0.56, 1.16)	0.71 (0.49, 1.02)	0.68 (0.47, 0.99)	0.71 (0.45, 1.13)	0.95 (0.66, 1.37)	
Diabetes								0.181
No	1768	Ref.	0.80 (0.55, 1.16)	0.90 (0.62, 1.31)	0.82 (0.56, 1.22)	0.90 (0.57, 1.43)	0.87 (0.59, 1.29)	
Yes	416	Ref.	0.82 (0.44, 1.50)	0.60 (0.32, 1.13)	0.55 (0.31, 0.99)	0.70 (0.31, 1.59)	1.15 (0.64, 2.07)	
Heart Failure								0.441
No	2045	Ref.	0.84 (0.59, 1.19)	0.91 (0.64, 1.29)	0.77 (0.54, 1.11)	0.79 (0.51, 1.24)	0.96 (0.67, 1.39)	
Yes	139	Ref.	0.69 (0.29, 1.62)	0.63 (0.29, 1.36)	0.53 (0.22, 1.23)	0.76 (0.28, 2.09)	1.10 (0.50, 2.42)	
Cardiovascular Disease								0.613
No	1824	Ref.	0.79 (0.53, 1.17)	0.79 (0.54, 1.17)	0.59 (0.39, 0.88)	0.87 (0.54, 1.40)	0.91 (0.61, 1.36)	
Yes	360	Ref.	0.92 (0.51, 1.67)	0.84 (0.47, 1.50)	0.92 (0.51, 1.64)	0.68 (0.31, 1.47)	1.03 (0.57, 1.84)	
Stroke								0.115
No	2037	Ref.	0.84 (0.59, 1.20)	0.84 (0.59, 1.19)	0.76 (0.53, 1.08)	0.97 (0.63, 1.49)	0.96 (0.67, 1.38)	
Yes	147	Ref.	0.43 (0.19, 0.94)	0.46 (0.21, 0.99)	0.28 (0.10, 0.81)	0.28 (0.09, 0.82)	0.76 (0.37, 1.57)	

Adjusted for gender, age, race, education level, ratio of family income to the poverty, muscle strengthening activities, smoking, hypertension, diabetes, heart failure, coronary heart disease and stroke (in addition to the stratification factor itself).

### Analysis of the combined effect of apple and banana intake

**Figure 5:**
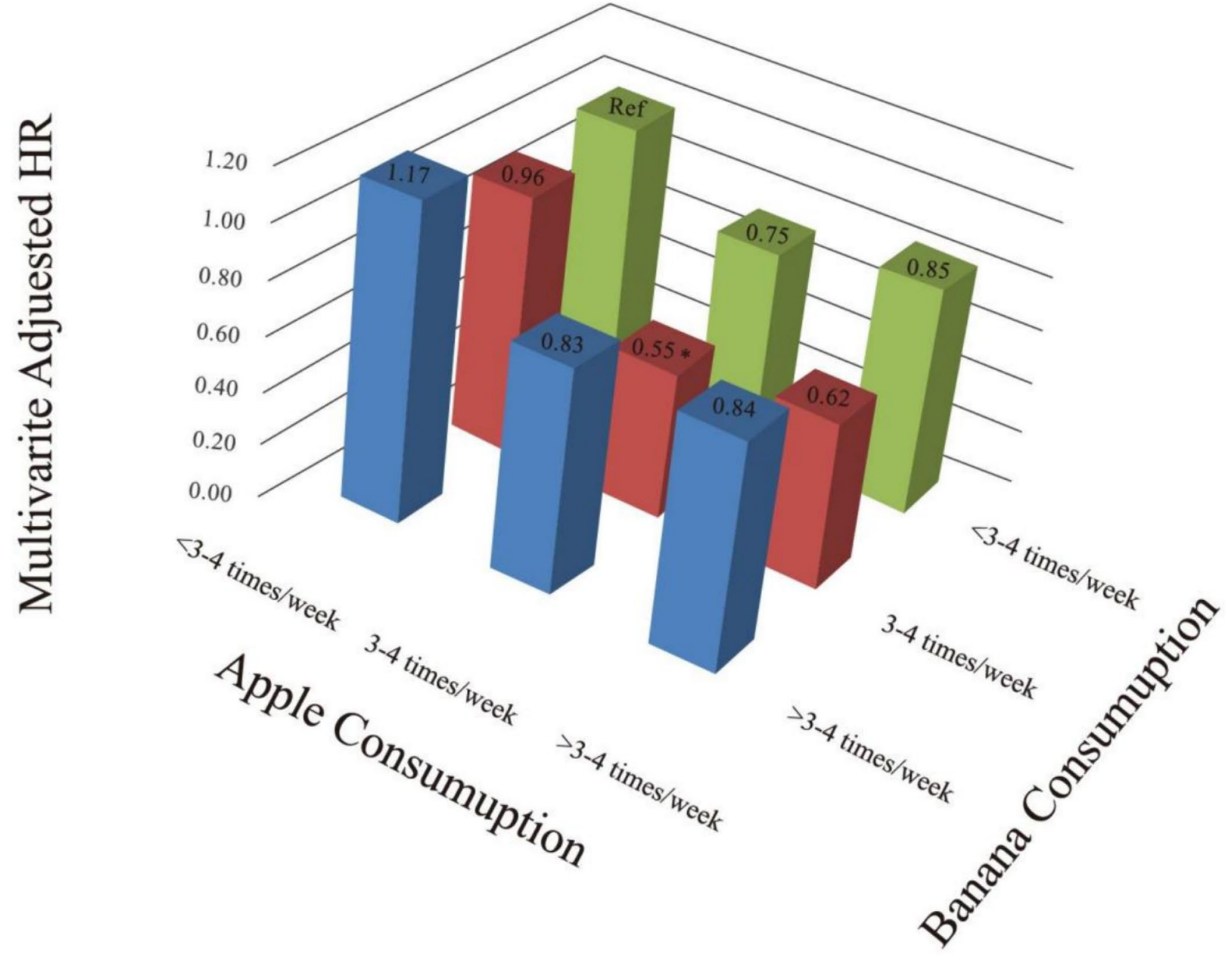
Three-dimensional histogram (combined consumption of apple and banana). Adjusted for gender, age, race, education level, ratio of family income to the poverty, muscle strengthening activities, smoking, hypertension, diabetes, heart failure, coronary heart disease and stroke.

Further analysis of the combined effect of apple and banana consumption on mortality in patients with dyslipidemia revealed that consuming both apples and bananas 3–4 times/week significantly reduced the 10-year all-cause mortality risk (HR = 0.55, 95% CI: 0.30–0.99, *p* = 0.045; Figure 5; Supplementary Table 1).

### Relationship between fruit consumption and cardiovascular and cerebrovascular disease mortality in patients with dyslipidemia

The impact of fruit consumption on death due to cardiovascular and cerebrovascular diseases was investigated using a competitive risk model. The results suggest that consuming apples 3–4 times/week can significantly reduce the risk of death due to cardiovascular and cerebrovascular diseases in patients with dyslipidemia (SHR = 0.46, 95%CI: 0.22–0.95, *p* = 0.035), consuming bananas 3–4 times/week does not reduce the risk of death due to cardiovascular and cerebrovascular diseases, but it can significantly reduce the risk of death due to other causes in patients with dyslipidemia (SHR = 0.64, 95%CI: 0.42–0.97, *p* = 0.036; Tables 5, 6).

**Table 5:** Competing risk models (apple consumption).

	SHR(95%CI)P	
Apple	Death of cardiovascular and cerebrovascular diseases	Death of All other cause
**<**1 times/month	Ref.	Ref.
1–3 times/month	1.03(0.67, 1.57)0.895	0.84(0.61, 1.17)0.305
1–2 times/week	0.68(0.42, 1.11)0.123	0.82(0.57, 1.18)0.284
3–4 times/week	0.46(0.22, 0.95)0.035	0.77(0.51, 1.16)0.213
5–6 times/week	1.10(0.51, 2.40)0.801	0.56(0.25, 1.29)0.175
≧1 times/day	0.85(0.42, 1.73)0.651	0.73(0.40, 1.32)0.293

**Table 6:** Competing risk models (banana consumption).

	SHR(95%CI)P	
Banana	Death of CVD	All other cause death
<1 times/month	Ref.	Ref.
1–3 times/month	1.02(0.59, 1.78)0.938	0.80(0.54, 1.19)0.275
1–2 times/week	0.61(0.33, 1.10)0.100	0.98(0.67, 1.45)0.934
3–4 times/week	1.03(0.60, 1.77)0.902	0.64(0.42, 0.97)0.036
5–6 times/week	0.94(0.48, 1.85)0.863	0.80(0.47, 1.36)0.418
≧1 times/day	0.98(0.56, 1.70)0.936	0.92(0.61, 1.41)0.714

## Discussion

The results of this study suggest that consuming apples or bananas 3–4 times/week significantly improves 10-year survival in patients with dyslipidemia and that consuming both apples and bananas is even more effective. However, further increasing the frequency of intake did not provide additional benefits. Based on the available literature, this is the first study to examine the effect of consuming multiple types of fruit on the risk of all-cause mortality in patients with dyslipidemia.

Past studies have linked fruit intake to reduced risks of obesity, dyslipidemia, diabetes mellitus, hypertension, and cardiovascular events (10–13). Some researchers have suggested that increased fruit intake can reduce the risk of chronic diseases, such as hypertension and cardiovascular disease (14). The benefits of fruit do not stop there; studies have demonstrated that fruits can regulate the intestinal microbiota, prevent inflammation, enhance the immunity of the intestinal mucosa, maintain the intestinal microecology, protect intestinal health, and prevent digestive disorders, such as constipation, irritable bowel syndrome, inflammatory bowel disease, and diverticulosis (15). Furthermore, fruits can reduce the severity of asthma (16) and the risks of chronic obstructive pulmonary disease and lung cancer (17). In addition to their physical health benefits, fruits can also promote mental health and reduce the risk of depression (18). Studies have demonstrated that fruits and vegetables do not only reduce the incidence of various diseases but also that their intake is negatively correlated with all-cause and cause-specific mortality from cancer and cardiovascular and respiratory diseases (19). A prospective study demonstrated that fruit consumption >42.9 g per day reduced all-cause mortality, cardiovascular disease mortality, and stroke mortality in patients with type 2 diabetes mellitus, compared with no fruit consumption (20). Additionally, increased fruit intake reduces the risk of death from ovarian and prostate cancer (21). One study noted that increased apple intake significantly reduced the risk of cardiovascular mortality (22). Another study pointed out that a moderate intake of apples and bananas reduced the mortality risk in patients with hypertension (9). By analyzing the data from NHANES, the present study demonstrated that consuming apples and bananas 3–4 times/week significantly reduced the risk of all-cause mortality in patients with dyslipidemia. This finding is consistent with the findings of past studies. Interestingly, apples and bananas appear to play different roles. Competitive risk model results suggest that consuming apples 3–4 times/week can significantly reduce the risk of death due to cardiovascular and cerebrovascular diseases, while consuming bananas 3–4 times/week does not reduce the risk of death due to cardiovascular and cerebrovascular diseases, but can significantly reduce the risk of death due to other causes in patients with dyslipidemia.

Recognized as a healthy way of eating, the Mediterranean diet reduces the incidence of cardiovascular disease, cancer, and diabetes mellitus (23) and may reduce cognitive decline and the risk of Alzheimer’s disease (24). The Mediterranean diet is a plant-based diet. Specifically, the Mediterranean diet is a plant-based dietary pattern characterized by plenty of fruits, vegetables, legumes and complex carbohydrates (whole grains), and monounsaturated fatty acids (25). However, the Mediterranean diet does not recommend consumption of specific types of fruits.

Fruits enhance health because of the antioxidant effects of vitamins C and E, as well as being rich in other nutrients including niacin, thiamin, riboflavin, vitamin B12, iron, potassium, and magnesium (26). In addition, apples and bananas have a high fiber content, and increasing dietary fiber intake may also reduce blood lipid levels, blood pressure, blood glucose levels, and cardiovascular risk (27, 28).

Apples are the third most produced fruit in the world (29), accounting for 12.5% of all fruit consumed in the United States. Apples, particularly unpeeled apples, are rich in polyphenols and fiber (30), with a content of 290 mg phenolic compounds per 100 g unpeeled apples, which is an antioxidant value equivalent to 1,500 mg vitamin C (31). Apple polyphenols are a major source of flavonoids, which not only reduce the accumulation of inflammatory cells but also reverse oxidative stress by inhibiting the expression of proinflammatory cytokines (interleukin-1 and tumor necrosis factor-*α*) through the p38 mitogen-activated protein kinase (MAPK) signaling pathway (32). Reactive oxygen species (ROS) are metabolic byproducts of biological systems, and they include superoxide radicals, hydrogen peroxide, and hydroxyl radicals (33). ROS can lead to endothelial dysfunction and promote inflammatory processes in monocytes, macrophages, or T-lymphocytes, as well as platelet aggregation, resulting in the development of atherosclerosis (34). Polyphenols may diminish ROS production by inhibiting oxidative enzymes, reducing superoxide production, inhibiting LDL formation, and ameliorating mitochondrial oxidative stress (35). Polyphenols, which have proven antioxidant properties, can modulate various cellular pathways and signaling cascades, lower serum cholesterol, raise high-density lipoprotein cholesterol levels, inhibit LDL oxidation, activate endothelial nitric oxide synthase, prevent platelet aggregation, and block inflammatory responses in atherosclerosis (30). A study analyzing the effect of apple polyphenols on atherosclerosis in mice demonstrated that apple polyphenols prevented oxidized LDL from inducing the MAPK/nuclear factor-κB activation pathway and reduced subsequent endothelial inflammation, which helped prevent the development of atherosclerosis (36). Reportedly, by interacting with intestinal microbes, polyphenols increase the bioavailability of polyphenols, promote the production of polyphenol metabolites, such as bile acids and short-chain fatty acids, and regulate lipid metabolism (33)while delaying lipid absorption in the intestine through changing the structure of the intestinal microbiota (37). A study using porcine and IPEC-J2 cell models indicated that apple polyphenols improve the intestinal mechanical barrier by enhancing intestinal antioxidant capacity and promoting the morphology and expression of intestinal tight junction proteins through the Nrf2/Keap1 signaling pathway (38). The study also demonstrated that polyphenols can improve intestinal immune function by increasing the number of intestinal microbiota, such as *Bifidobacterium* and *Lactobacillus*, and intestinal immunoglobulin A levels (38). The main soluble dietary fiber in apples is pectin, which can regulate the intestinal microbiota and influence gastric emptying time and nutrient absorption, thus affecting lipid and glucose metabolism (30). The ability of apples to lower serum cholesterol is mainly attributed to polyphenols and soluble fiber, which may have a synergistic effect (39). The high dietary fiber and polyphenol content, as well as the hypolipidemic and antioxidant properties, of apples have made them a food of choice for the prevention of cardiovascular disease (40).

Banana is the fruit of an evergreen monocotyledonous, perennial, giant herbaceous plant that grows mainly in tropical and subtropical regions (41). Banana contains a variety of bioactive compounds, such as phenolics, carotenoids, biogenic amines, and phytosterols. Flavonols are the main constituents of heavy phenolic compounds with potent antioxidant effects in bananas (42). Flavonoids act as antioxidants, scavenging free radicals and ROS, and reduce total cholesterol levels by increasing the degradation and excretion of bile acids and neutral sterols (43). Carotenoids have the unique ability to scavenge singlet oxygen, thereby protecting cell membrane lipids from free radicals. The consumption of carotenoid-rich foods can boost immunity and reduce the risks of cancer, diabetes mellitus, and heart disease (44). The biogenic amines in bananas include serotonin, dopamine, and norepinephrine, which contribute to a sense of happiness. Phytosterols can lower blood cholesterol levels and reduce their absorption in the intestine (45).

Apples and bananas are not the only fruits rich in polyphenols and fiber; many other fruits are also rich in these nutrients. However, this study demonstrated that pineapple and grapes did not have a similar protective effect as that of apples and bananas in patients with dyslipidemia. Apples, pears, and peaches have similar fiber contents; however, apples contain a higher level of phenolic compounds (46). Therefore, apples are more effective in lowering cholesterol (40). Anthocyanins and proanthocyanidins in apples are absorbed more efficiently in the human body than are those in grapes (47), and apples contain more dietary fiber than grapes do. Pineapples are rich in dietary fiber, phenolic compounds, and flavonoids, and have antioxidative properties (48). However, the predominant fiber components in pineapple are hemicellulose and cellulose (49), which are insoluble fibers, and these fibers are essentially unaltered during digestion (4). Moreover, the polyphenol content of pineapple is approximately five times lower than that of banana (50).

In the United States, at least 100 million adults have dyslipidemia, which is a major contributor to the high incidence of cardiovascular events. Early intervention for dyslipidemia plays an important role in primary prevention of cardiovascular diseases, preventing up to 80% of cardiovascular disease-related deaths (51). American Heart Association guidelines emphasize the importance of establishing a healthy lifestyle, including appropriate diet and exercise (52). Research has shown that the Mediterranean diet can significantly reduce the incidence of cardiovascular diseases (53). Many patients fail to recognize the importance of a healthy diet or devote sufficient attention to needed dietary changes, and prescribed diets often involve complicated operational procedures. Many patients thus cannot strictly follow medically sound dietary advice. According to previous studies, few people adhere to effective dietary patterns in their daily lives (54). Increasing the intake of fruits in patients with dyslipidemia is a well-recognized dietary improvement; however, there is no standard recommendation for specific fruit types or their daily servings. This study provides guidance for patients with dyslipidemia when choosing fruit, and provides a specific intake frequency. This study included many types of fruit. Pineapples and grapes did not improve the prognosis of patients with dyslipidemia (*p* > 0.05). Only apples and bananas significantly reduced the risk of all-cause mortality in patients with dyslipidemia. It is particularly noteworthy that apple consumption 3–4 times/week can significantly reduce the risk of death due to cardiovascular and cerebrovascular diseases in patients with dyslipidemia. Apples and bananas are widely consumed around the world, and improving dietary patterns with these fruits is an economical and effective way for the early intervention of dyslipidemia. Clinical workers or nutritionists can recommend fruits (such as apples and bananas) to improve the prognosis of patients with dyslipidemia and, on this basis, formulate more personalized and specific dietary intervention programs according to patients’ personal preferences, economic conditions, and eating habits.

## Limitations

This study has some limitations. The research population of this study was from NHANES, so the results of this study are only applicable to the American population. Whether the results can be extended to other countries remains to be confirmed by future studies. Due to limited information in the NHANES database, a wide range of fruits were excluded from this study. We cannot deny the health-promoting properties of other fruits, thus various kinds of fruit need to be examined in future studies. Considering the possible influence of differences in fruit morphology (such as whole fruit or juice) on the results, it is necessary to confirm whether different forms of fruit products have the same effect in the future. Although we have tried to include all confounding variables that may affect the results and reduce the influence of covariates in our analyses, uncertainties and unmeasured factors may still affect our research results.

## Conclusion

The present study demonstrated that a moderate intake of apples or bananas was effective in reducing the mortality risk in patients with dyslipidemia and that consuming both fruits led to greater benefits. This finding facilitates the formulation of more favorable dietary recommendations for patients with dyslipidemia.

## Data Availability

Publicly available datasets were analyzed in this study. This data can be found at: https://www.cdc.gov/nchs/nhanes/index.htm.

## Footnotes

^1^https://www.cdc.gov/nchs/nhanes/index.htm

^2^http://www.empowerstats.com
