# Supplementary material for: Correlation between fruit consumption and 10-year all-cause mortality in patients with dyslipidemia
Source: Front Nutr. 2024 Oct 3;11:1471737. doi: 10.3389/fnut.2024.1471737 (PMC11484278; doi:10.3389/fnut.2024.1471737)
Supplement: Supplementary file 1 [file Table_1.doc]

**Supplementary Table 1. Cox regression analysis（Combined consumption of apple and banana）**

| **Fruit consumuption** | | **Banana** | | |
| --- | --- | --- | --- | --- |
|  |  | **HR(95%CI)P** | | |
|  |  | ＜3-4 times/month | **3-4 times/week** | ＞3-4 times/month |
| **Apple** | **＜3-4 times/month** | Ref. | 0.96 (0.71, 1.30) 0.792 | 1.17 (0.89, 1.53) 0.254 |
|  | **3-4 times/week** | 0.75 (0.42, 1.35) 0.336 | 0.55 (0.30, 0.99) 0.045 | 0.83 (0.47, 1.47) 0.522 |
|  | **＞3-4 times/month** | 0.85 (0.44, 1.67) 0.646 | 0.62 (0.25, 1.51) 0.294 | 0.84 (0.54, 1.32) 0.454 |

Adjusted for gender, age, race, education level, ratio of family income to the poverty, muscle strengthening activities, smoking, hypertension, diabetes, heart failure, coronary heart disease and stroke.

**Supplementary Table 2. Cox regression analysis**

|  | **HR(95%CI)P** | | | |
| --- | --- | --- | --- | --- |
|  | **Apple** | **Banana** | **Pineapple** | **Grape** |
| **﹤**1 times/month | Ref. | Ref. | Ref. | Ref. |
| **1-3 times/month** | 0.91 (0.71, 1.18) 0.481 | 0.82 (0.59, 1.12) 0.211 | 0.93 (0.73, 1.19) 0.584 | 0.87 (0.69, 1.09) 0.212 |
| **1-2 times/week** | 0.77 (0.58, 1.02) 0.065 | 0.78 (0.57, 1.07) 0.121 | 0.95 (0.62, 1.45) 0.810 | 1.07 (0.80, 1.44) 0.636 |
| **3-4 times/week** | 0.61 (0.42, 0.87) 0.006 | 0.71 (0.52, 0.98) 0.039 | 1.17 (0.48, 2.85) 0.728 | 0.87 (0.53, 1.41) 0.560 |
| **5-6 times/week** | 0.67 (0.39, 1.17) 0.159 | 0.79 (0.53, 1.17) 0.236 | 1.25 (0.17, 8.99) 0.823 | 0.54 (0.27, 1.11) 0.092 |
| **≧**1 times/day | 0.77 (0.49, 1.19) 0.235 | 0.95 (0.69, 1.32) 0.778 | 0.73 (0.25, 2.07) 0.549 | 0.65 (0.31, 1.32) 0.233 |

Adjusted for gender, age, race, education level, ratio of family income to the poverty, muscle strengthening activities, alcohol use, smoking, hypertension, diabetes, heart failure, coronary heart disease and stroke.
